# Infraorbital cutaneous angiosarcoma: a diagnostic and therapeutic dilemma

**DOI:** 10.1186/1746-160X-4-18

**Published:** 2008-08-11

**Authors:** Tobias Ettl, Johannes Kleinheinz, Ravi Mehrotra, Stephan Schwarz, Torsten E Reichert, Oliver Driemel

**Affiliations:** 1 Department of Oral and Maxillofacial Surgery, Regensburg University, Germany; 2 Department of Oral and Maxillofacial Surgery, Muenster University, Germany; 3 Department of Pathology, Moti Lal Nehru Medical College, Allahabad University, India; 4 Department of Pathology, Erlangen University, Germany

## Abstract

**Background:**

A cutaneous angiosarcoma is a rare malignant tumour of vascular endothelial cells with aggressive clinical behaviour and poor prognosis. Diagnosis is often delayed due to its variable and often benign clinical appearance.

**Case presentation:**

This case presents a 64-year-old man with a six-month-history of a recurrent diffuse and erythematous painless swelling below the left eye. Several resections with intraoperatively negative resection margins followed, but positive margins were repeatedly detected later on permanent sections. Histopathologic examination of the specimen diagnosed a cutaneous angiosarcoma. Neither, finally achieved negative margins on permanent sections, nor a following chemotherapy could prevent the recurrence of the disease after five months and the patient's dead 21 months after the first diagnosis.

**Conclusion:**

The case elucidates the current diagnostic and therapeutic dilemma of this entity, which shows an unfavourable clinical course in spite of multimodal therapy.

## Background

A cutaneous angiosarcoma (synonyms: lymphangiosarcoma and haemangiosarcoma) is a rare malignant tumour of vascular endothelial cells. It occurs predominantly in the elderly and is confined to the face and the scalp region in more than 50% of cases [1]. Despite the aggressive behaviour and poor prognosis, the diagnosis is often delayed due to its variable and often benign clinical appearance. This case documents a facial cutaneous angiosarcoma in an elderly male patient, revealing the diagnostic and therapeutic dilemma of this entity, which shows an unfavourable clinical course in spite of multimodal therapy.

## Case report

**Figure 1:**
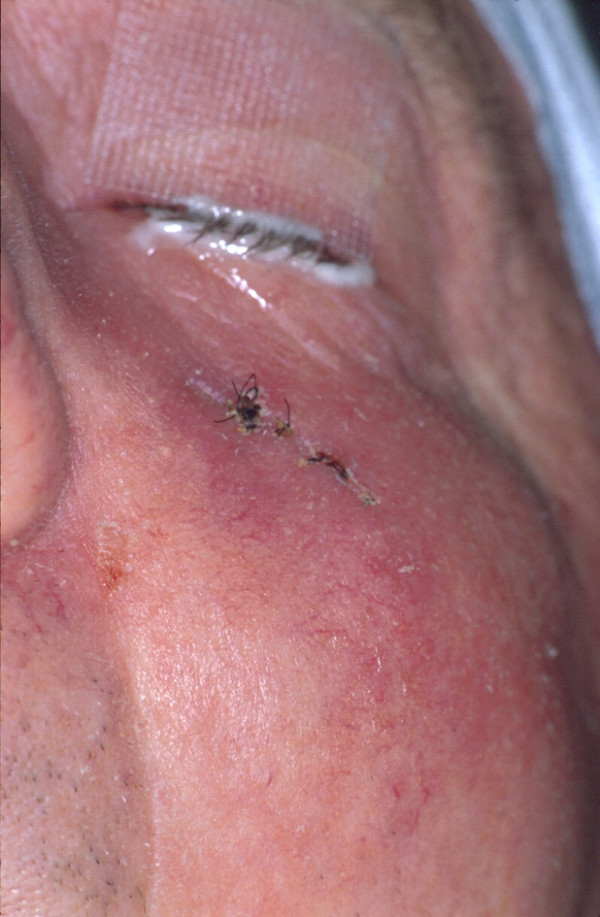
Clinical appearance after first incisional biopsy: Discreet skin erythema below the left eye.

**Figure 2:**
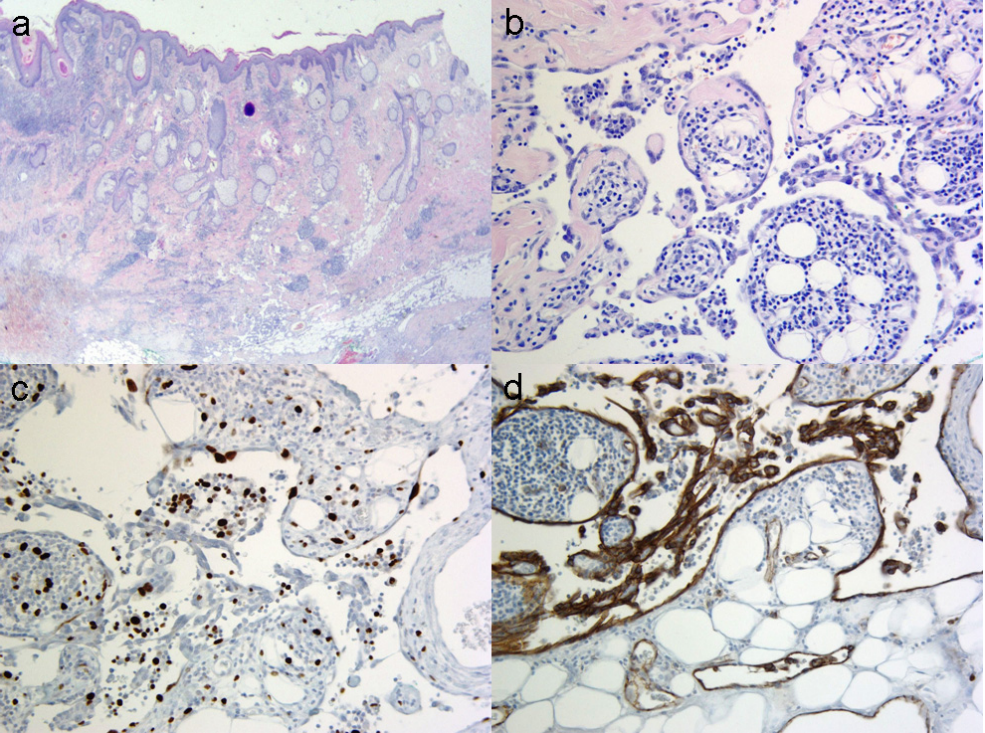
**Histopathology.** a: Overview image: Epidermis, followed by dermis with hair follicles and sebaceous glands. Tumour with unclear borders in the depth (H&E, 16×). b: In detail: Atypic, swollen endothelial cells with anastomosing, pseudopapillar patterns and lymphocytic inflammation (H&E, 200×). c: Immunohistochemistry with proliferation marker MIB-1 indicating proliferation in about 5%–10% of the cells (MIB-1, 200×). d: Positive immunohistochemical reaction to the endothelial marker CD 31 (CD 31, 200×).

A 64-year-old man presented with a six month history of a recurrent diffuse and erythematous painless swelling (3 × 2 cm^2^) below the left eye to the Department of Dermatology, Regensburg University, Germany. Cervical lymphadenopathy was clinically not detectable. Routine laboratory results showed no abnormality. Presuming an allergic dermatitis, topical treatment with steroids was initiated. Because of the persistence of the lesion, an incisional biopsy was performed three weeks later (Figure 1). Histopathology of the specimen showed an invasively growing tumour of the dermis, composed of atypical vascular endothelia in a disordered manner, forming bizarre vascular lumina. The tumor cells were characterized by an elevated proliferated activity with a proliferation fraction (MIB-1) of 5%–10%. The vascular endothelial proliferation showed a papillary architecture accompanied by small lymphocytes. The majority of endothelial cells presented a hyperchromatic nucleus and a swollen cytoplasm. (Figure 2a, 2b, 2c). Immunohistochemical studies demonstrated positivity for CD 31 (Figure 2d) and factor VIII-related antigen. Based on these findings the diagnosis of a cutaneous angiosarcoma was made.

**Figure 3:**
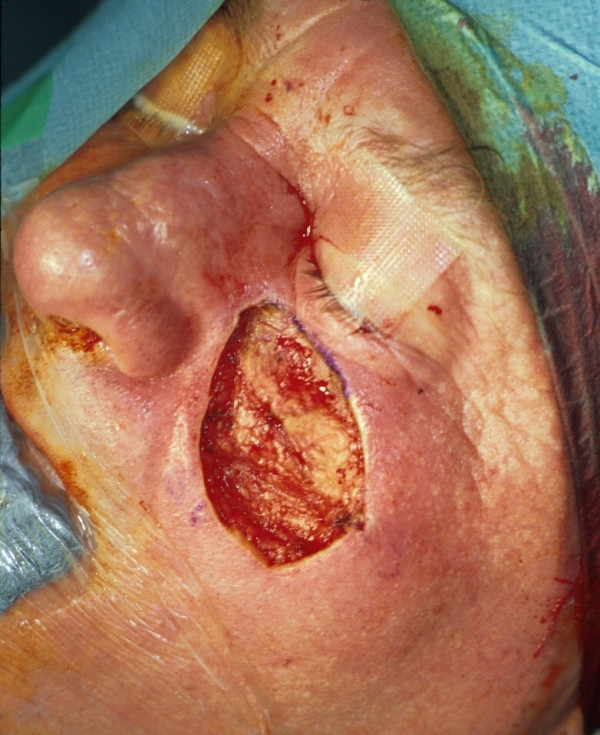
Clinical finding after first surgery: Intraoperative defect, 4 × 2.5 cm^2 ^in size.

After referral of the patient to the Department of Oral and Maxillofacial Surgery, Regensburg University, Germany, the tumour was removed by wide local surgical excision (Figure 3) and the defect was temporarily covered by Epigard. Despite negative intraoperative frozen section margins, positive margins were repeatedly detected later on permanent sections. Negative margins on permanent section were finally reached after three resections and infraorbital soft tissue was plastically reconstructed with a buccal rotation flap. After surgery, chemotherapy followed with six cycles of alpha-interferon.

**Figure 4:**
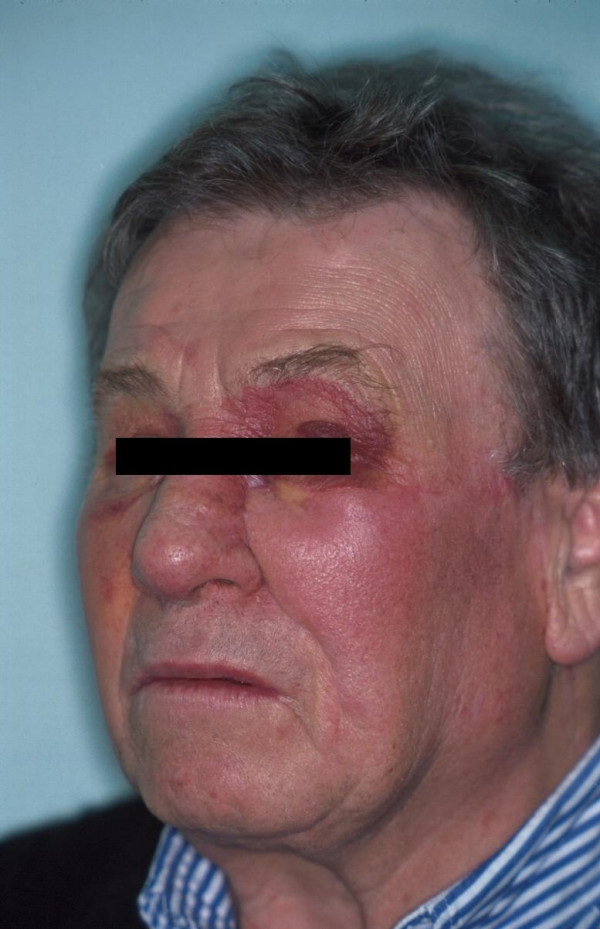
Recurrence 5 months after first surgery: Periorbital erythema and swelling on both sides (left more than right).

**Figure 5:**
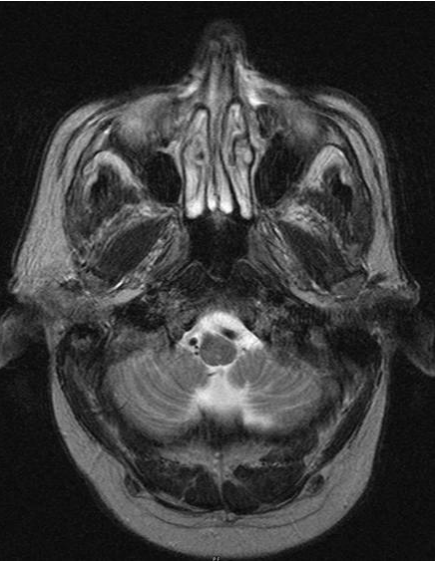
MRI (axial): Left infraorbital mass with infiltration to the lateral nasal root.

Five months later a periorbital redness and swelling on both sides (Figure 4) required another incisional biopsy, which was confirmed as recurrent angiosarcoma on histopathological examination. Imaging staging procedures (MRI and CT head-neck, CT chest, CT abdomen, PET and bone scan) found bone invasion to the nasal root (Figure 5). Metastases to the neck lymph nodes as well as distant metastases were clinically and radiologically excluded.

Neither radiochemotherapy with a cumulative radiation dose of 64.8 Gy and seven cycles Doxorubicin nor an additional antiangiogenetic therapy with Trofosfamide, Pioglitazone, Rofecoxibe and steroids could prevent the rapid tumour progression. The patient died 21 months after the first diagnosis.

## Discussion

There are three main types of cutaneous angiosarcoma: Idiopathic angiosarcoma of the head and neck in elderly patients, lymphoedema-associated angiosarcoma (Stewart-Treves-Syndrome) and postirradiation angiosarcoma [2]. Besides an association with persistent chronic lymphoedema, previous irradiation and pre-existing vascular malformation, little is known regarding the causative factors of that disease [3]. With respect to pathogenesis, among others, upregulation of the glykopeptide VEGF-D, a vascular endothelial growth factor, seems to be responsible for the endothelial cell proliferation [4].

Clinically the appearance of a cutaneous angiosarcoma of the skin and scalp can be variable. Early lesions most commonly present as single or multifocal ill-defined, bruise-like erythematous-purplish areas with indurated borders [5]. In the present case, akin to those previously described by others [6,7], these haematoma-like lesions can be misinterpreted as benign inflammatory or allergic hyperemias. More advanced lesions can present as dark bluish, sometimes keratotic papules or nodules with ulceration and bleeding, mimicking other malignancies like squamous cell carcinoma, basal cell carcinoma, malignant melanoma, lymphoma as well as metastases [3,5,8].

Microscopically a cutaneous angiosarcoma is typically characterized by numerous, irregular and anastomosing vascular channels. These are lined by pleomorphic, hyperchromatic endothelial cells with variable mitotic activity [9]. Immunhistochemical positivity for the endothelial markers CD 31 and factor VIII-related antigen as well as for the transcription factor Fli-1 may help to establish diagnosis [10,11]. The differential diagnosis includes hemangioma, especially tufted, cavernous and epithelioid hemangioma on the one hand and acantholytic carcinoma on the other hand. Especially in immunocompromised patients Kaposi-sarcoma might be a further differential diagnosis. In the current case the presence of many lymphocytes might be a hint to regard the lesion as of lymphatic vessel origin, i.e. as a lymphangiosarcoma.

Treatment of the cutaneous angiosarcoma is generally based on radical surgery and postoperative radiation therapy. Surgery is postulated to attain a wide excision of the tumour with histologically negative margins [1,4]. Unfortunately achieving negative margins is difficult, as multifocal and extensive microscopic spread is common in this disease. Intraoperative frozen sections are often performed to assist in determining section margins. Pawlik et al. [5] demonstrated, however, an overall negative predictive value of only 33.3% for that procedure, which explains the repeating surgical resections in the case report. For this reason, temporary reconstruction with homografts or skin substitutes is recommended until the definite histological confirmation of margin status. Since up to 78% of the patients still have residual tumour after wide and multiple surgical resections [5,12], this goal of achieving histologically negative section margins remains debatable. In many cases the resulting extensive resection defects require large secondary plastic reconstruction.

More recently, chemotherapy and gene therapy are increasingly available. Doxorubicin is reported to be active in angiosarcoma [13], but did not show response in the present patient. Paclitaxel is another agent, that seems to have substantial effects, even in patients, who were treated previously with chemotherapy or radiation therapy [2,14]. In more palliative situations, antiangiogentic therapy with pioglitazone, rofecoxib and metronomic trofosfamide has been recommended [15].

## Conclusion

Despite multimodal therapy options, prognosis of the cutaneous angiosarcoma is still poor, with a 5-year-survival rate between 12% and 33%. About half of the patients are dying within 15 to 18 months of presentation [1,5,16]. The most important positive prognostic factors seem to be young age, small tumour size, negative resection margins and radiation therapy [3,5,8].

In summary, the present case of a cutaneous angiosarcoma of the face elucidates the current diagnostic and therapeutic dilemma of this lesion. Diagnosis is often delayed, due to its putatively innocous clinical appearance. Negative microscopic section margins are hardly achieved during surgery, resulting in multiple operations with large postoperative defects. Despite multimodal therapy concepts, the prognosis remains poor.

## Competing interests

The authors declare that they have no competing interests.

## Authors' contributions

TE drafted the manuscript. JK helped to the critical review of the article. RM helped to the critical review of the article. SS performed the histopathological investigations. TER helped to the critical review of the manuscript. OD performed the surgical procedure, helped to draft the manuscript, helped to the critical review of the manuscript.

All authors read and approved the final manuscript.

## Consent section

Written informed consent was obtained from the patient for publication of this case report and accompanying images. A copy of the written consent is available for review by the Editor-In-Chief of this journal.
